# Correction: Atomic-scale detection of individual lead clusters confined in Linde Type A zeolites

**DOI:** 10.1039/d2nr90249d

**Published:** 2023-01-11

**Authors:** Jarmo Fatermans, Giacomo Romolini, Thomas Altantzis, Johan Hofkens, Maarten B. J. Roeffaers, Sara Bals, Sandra Van Aert

**Affiliations:** a Electron Microscopy for Materials Science (EMAT), University of Antwerp Groenenborgerlaan 171 2020 Antwerp Belgium sandra.vanaert@uantwerpen.be; b NANOlab Center of Excellence, University of Antwerp Belgium sandra.vanaert@uantwerpen.be; c Molecular Imaging and Photonics, Department of Chemistry, KU Leuven Celestijnenlaan 200F 3001 Leuven Belgium; d Applied Electrochemistry and Catalysis Group (ELCAT), University of Antwerp Universiteitsplein 1 2610 Wilrijk Belgium; e Max Planck Institute for Polymer Research Ackermannweg 10 55128 Mainz Germany; f Centre for Membrane Separations, Adsorption, Catalysis, And Spectroscopy for Sustainable Solutions (cMACS), KU Leuven Celestijnenlaan 200F Box 2461 3001 Leuven Belgium maarten.roeffaers@kuleuven.be

## Abstract

Correction for ‘Atomic-scale detection of individual lead clusters confined in linde type A zeolites’ by Jarmo Fatermans *et al.*, *Nanoscale*, 2022, **14**, 9323–9330, https://doi.org/10.1039/D2NR01819E.

The authors wish to update their Acknowledgements to recognise the European Synchrotron Radiation Facility. The full and correct Acknowledgements can be found below:

“The authors acknowledge the Research Foundation Flanders through project fundings (FWO, G026718N, G050218N, ZW15_09-G0H6316N, and W002221N) and through a PhD scholarship to G. R. (grant 11C6922N), as well as iBOF-21-085 PERSIST. T. A. and S. V. A. acknowledge funding from the University of Antwerp Research fund (BOF). J. H. acknowledges the Flemish government through long-term structural funding Methusalem (CASAS2, Meth/15/04) and the MPI as MPI fellow. M. R. acknowledges funding by the KU Leuven Research Fund (C14/19/079). S. B. and S. V. A. acknowledge funding from the European Research Council under the European Union's Horizon 2020 Research and Innovation Program (ERC Consolidator Grants No. 815128 – REALNANO and No. 770887 – PICOMETRICS). We acknowledge the European Synchrotron Radiation Facility for the provision of synchrotron radiation facilities, and we would like to thank Dr D. Chernyshov for assistance in using beamline BM01”.

This new text supersedes the Acknowledgements originally provided with the article.

The Royal Society of Chemistry apologises for these errors and any consequent inconvenience to authors and readers.

## Supplementary Material

